# Mortality trends in the United States in recent years including provisional 2024 data

**DOI:** 10.1016/j.pmedr.2026.103373

**Published:** 2026-01-03

**Authors:** Shehroze Tabassum, Faraz Azhar, Abdul Mannan Khan Minhas, Dmitry Abramov

**Affiliations:** a Department of Internal Medicine, The Wright Center for Graduate Medical Education, Scranton, PA, USA; b Department of Internal Medicine, Allama Iqbal Medical College, Lahore, Pakistan; c Department of Medicine, Section of Cardiology, Baylor College of Medicine, Houston, TX, USA; d Division of Cardiology, Department of Medicine, Loma Linda University Health, Loma Linda, CA, USA

**Keywords:** COVID-19, Mortality, Cardiovascular disease, Epidemiology, CDC WONDER

## Abstract

**Objectives:**

The COVID-19 pandemic resulted in greater mortality in the United States. We sought to evaluate ongoing rates of all-cause and of top individual causes of mortality using provisional 2024 mortality data.

**Methods:**

Data on all-cause and top individual causes of mortality in the United States between 2018 and provisional 2024 estimates, using Age-Adjusted and Crude Mortality rates (with 95 % Confidence Intervals), were obtained from the publicly available Center for Disease Control and Prevention Wide-Ranging Online Data for Epidemiologic Research data set.

**Results:**

All-cause age-adjusted mortality rates 100,000 from 2018 and provisional 2024 data were 723.6 (722.8724.5), 715.3 (714.4716.1), 835.4 (834.5836.3), 879.7 (878.7880.6), 798.8 (797.9799.9), 750.5 (749.7751.4), and 742.6 (741.8743.5), with provisional 2024 rates remaining above pre-pandemic baseline. Mortality attributed to COVID-19 infection peaked in 2021 as the 3rd leading cause of death, dropping to the 4th, 11th, and 15th leading cause of death in 2022, 2023, and provisional 2024 data respectively.

**Conclusions:**

All-cause mortality rates in provisional 2024 data in the United States remain above pre-pandemic baseline. Mortality from COVID dropped from the 3rd leading cause of death in 2021 to the 15th leading cause of death in provisional 2024 data.

## Highlights

• Provisional US 2024 all-cause mortality estimates remain above pre-pandemic rates
• Mortality from COVID decreased to the 15th leading cause in 2024
• All-cause mortality rates in 2024 remained above pre-pandemic for most cohort.

## 1 Introduction

The COVID-19 pandemic, which started in 2020, led to a rise in all-cause mortality and a rise in many individual causes of mortality in the United States (Ahmad et al., 2024; Bor et al., 2025; Minhas et al., 2024). All-cause mortality peaked in 2021 and remained above the pre-pandemic baseline in 2022 and 2023, with higher rates of all-cause mortality seen across cohorts based on sex, race and ethnicity, and most age groups (Ahmad et al., 2024; Minhas et al., 2024). The pandemic also led to increases in mortality individual causes including cardiovascular disease, accidents, diabetes mellitus, renal diseases, among other top causes of mortality; with mortality from these conditions predominantly peaking in 2021 (Ahmad et al., 2024; Minhas et al., 2024). In the subsequent two years, all-cause mortality as well as mortality from several top causes of death declined. In provisional 2023 mortality data, mortality from some conditions, including diseases of the heart and suicide, appeared to return to pre-pandemic rates while mortality from other conditions, including diabetes mellitus, and chronic liver disease, remained significantly above pre-pandemic rates (Minhas et al., 2024). Regarding COVID mortality, prior analyses demonstrated that COVID mortality peaked in 2021 as the 3rd leading cause of death, dropping to the 4th leading cause in 2022, and further dropping to the 10th leading cause in 2023 (Minhas et al., 2024). To further characterize the evolving mortality trends associated with the COVID pandemic, we sought to evaluate trends in all-cause mortality and mortality from top causes of death in finalized 2023 data and provisional 2024 data as available from mortality files using the Center for Disease Control and Prevention Wide-Ranging Online Data for Epidemiologic Research. Evaluation of the latest available mortality data is important for public health efforts to understand the ongoing burden of mortality attributed to COVID itself as well as to understand the residual effects of the pandemic on other common causes of death.

## 2 Methods

### 2.1 Study design

Mortality data within the United States was obtained using Center for Disease Control and Prevention Wide-Ranging Online Data for Epidemiologic Research, with data output derived from the reporting software available on https://wonder.cdc.gov/wonder/help/mcd-provisional.html (*Provisional Mortality Statistics by Multiple Cause of Death and by Single Race, for 2018 through Present. https://wonder.cdc.gov/wonder/help/mcd-provisional.html*, 2024). Center for Disease Control and Prevention Wide-Ranging Online Data for Epidemiologic Research presents data compiled by the National Center for Health Statistics and contains mortality data on over 99 % of deaths in the United States (Vital Statistics of United States, 1999). As such, mortality data within Center for Disease Control and Prevention Wide-Ranging Online Data for Epidemiologic Research is considered to be the definitive source for United States mortality (Ahmad et al., 2021) and therefore presents an optimal opportunity to evaluate causes of death and mortality trends in the United States. Approval from an Institutional Review Board and informed consent were not necessary as the study utilized publicly accessible data that contained no identifiable personal information.

### 2.2 Populations

Mortality data overall, by sex, race and ethnicity, and age were collected for the years 2018 through 2024, with data for 2024 still considered provisional at the time of data collection in 4/2025. Information regarding the population data used as denominators is available in the dataset documentation (*Provisional Mortality Statistics by Multiple Cause of Death and by Single Race, for 2018 through Present. https://wonder.cdc.gov/wonder/help/mcd-provisional.html*, 2024). Race and ethnicity were categorized as Non-Hispanic White, Non-Hispanic Black or African American, Non-Hispanic Asian, Non-Hispanic Native Hawaiian or Other Pacific Islander, Non-Hispanic American Indian or Alaska Native, Non-Hispanic more than one race, and Hispanic or Latino, based on classifications available within Center for Disease Control and Prevention Wide-Ranging Online Data for Epidemiologic Research. Age groups were defined as under 1 year, 1–4 years, 5–14 years, 15–24 years, 25–34 years, 35–44 years, 45–54 years, 55–64 years, 65–74 years, 75–84 years, and 85 years and older, based on classifications available within Center for Disease Control and Prevention Wide-Ranging Online Data for Epidemiologic Research.

### 2.3 Measures

Top 15 underlying causes of deaths as characterized within the database for each study year were also extracted. Age-adjusted mortality rates (with 95 % Confidence Intervals) per 100,000 individuals were evaluated to allow better comparison across populations with different age structures over time while crude mortality rates (with 95 % Confidence Intervals) were evaluated when reporting mortality based on age cohort. Age-adjusted mortality rates were determined using 2000 U.S. standard population (Anderson and Rosenberg, 1998). Statistical differences in mortality rates between cohorts, years, or causes of death were inferred from non-overlapping confidence intervals.

## 3 Results

**Fig. 1:**
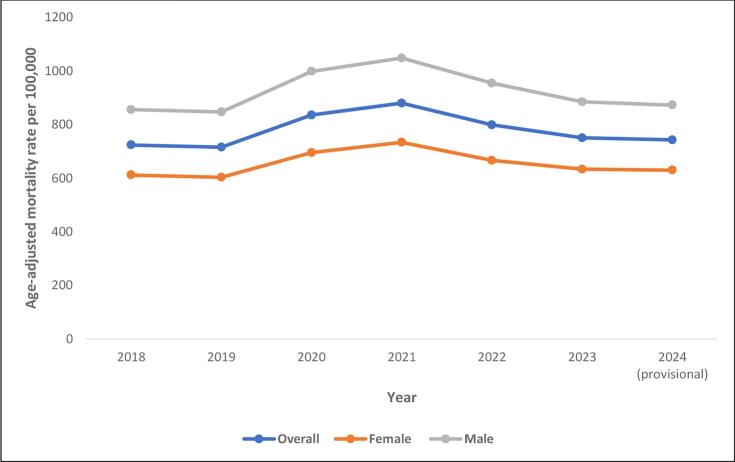

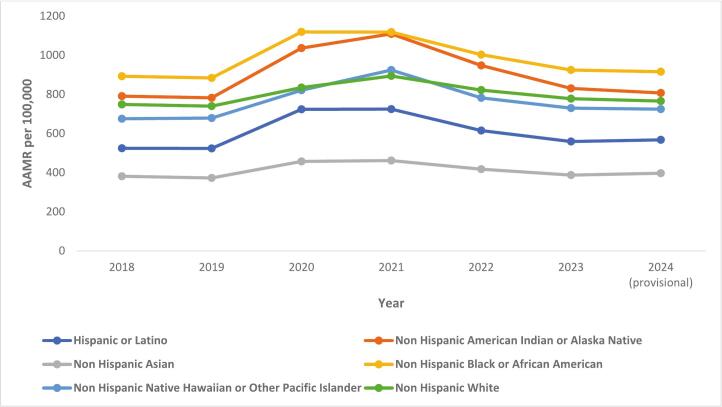
A Overall and sex stratified all-cause mortality trends in the United States obtained from Center for Disease Control and Prevention Wide-Ranging Online Data for Epidemiologic Research, 2018 to 2024. B All-Cause Mortality Trends stratified by race and ethnicity in the United States obtained from Center for Disease Control and Prevention Wide-Ranging Online Data for Epidemiologic Research, 2018 to 2024.

Between 2018 and 2024, all-cause age-adjusted mortality rates per 100,000 people were 723.6 (722.7724.5), 715.3 (714.4716.1), 835.4 (834.5836.3), 879.7 (878.7880.6), 798.8 (797.9799.9), 750.5 (749.7751.4), and 742.6 (741.8743.5), respectively. Similar patterns, with a mortality peak in 2021, were observed across sexes, racial and ethnic populations, and most age groups **(**Table 1**)**. In 2024, provisional all-cause age-adjusted mortality rates remained statistically higher than the levels recorded in 2019, prior to the pandemic **(**Fig. 1. A. A**)**. Provisional all-cause mortality in 2024 remained statistically higher than in 2019 for both sexes and all racial and ethnic populations, except potentially Non-Hispanic Native Hawaiian or Other Pacific Islander. Provisional 2024 mortality was statistically higher than 2019 mortality for some age groups except those under 1 year of age and those aged 15–24, 25–34, 45–54 and 55–64 (where provisional 2024 mortality was lower or similar to 2019 mortality). Mortality in 2024 remained highest in Non-Hispanic Black or African American individuals, followed by Non-Hispanic American Indian or Alaska Native, Non-Hispanic White, Non-Hispanic Native Hawaiian or Other Pacific Islander, Hispanics or Latino, Non-Hispanic Asian and Non-Hispanic more than one race individuals **(**Fig. B**).**

**Table 1:** All-Cause United States mortality, expressed as Crude Mortality Rates/Age-Adjusted Mortality Rates per 100,000 population, obtained from Center for Disease Control and Prevention Wide-Ranging Online Data for Epidemiologic Research data set from 2018 to 2024.

	**2018**		**2019**		**2020**		**2021**		**2022**		**2023**		**2024 (provisional)**	
	Age-Adjusted Mortality Rate	Age-Adjusted Mortality Rate (95 % CI)	Age-Adjusted Mortality Rate	Age-Adjusted Mortality Rate (95 % CI)	Age-Adjusted Mortality Rate	Age-Adjusted Mortality Rate (95 % CI)	Age-Adjusted Mortality Rate	Age-Adjusted Mortality Rate (95 % CI)	Age-Adjusted Mortality Rate	Age-Adjusted Mortality Rate (95 % CI)	Age-Adjusted Mortality Rate	Age-Adjusted Mortality Rate (95 % CI)	Age-Adjusted Mortality Rate	Age-Adjusted Mortality Rate (95 % CI)
Overall	723.6	722.8724.5	715.3	714.4716.1	835.4	834.5836.3	879.7	878.7880.6	798.8	797.9799.7	750.5	749.7751.4	742.6	741.8743.5
< 1 year	557.8	550.4565.3	553.0	545.5560.5	524.3	517.0,531.6	558.9	551.1566.6	558.0	550.5,65.7	552.1	544.6,59.8	543.1	535.5550.7
1–4 years	24.0	23.2,24.8	23.3	22.5,24.0	22.7	21.9,23.4	25.0	24.2,25.8	28.0	27.1,28.8	27.3	26.5,28.2	25.7	24.9,26.5
5–14 years	13.3	12.9,13.6	13.4	13.1,13.8	13.7	13.4,14.1	14.3	14.0,14.7	15.3	14.9,15.6	14.7	14.3,15.0	14.4	14.1,14.8
15–24 years	70.2	69.4,71.0	69.7	69.0,70.5	84.2	83.3,85.0	88.9	88.0,89.8	79.5	78.6,80.3	76.8	76.0,77.6	68.1	67.3,68.8
25–34 years	128.8	127.7129.8	128.8	127.8129.9	159.5	158.4160.7	180.8	179.6182.1	163.4	162.3164.6	148.1	147.0,149.2	126.6	125.6127.7
35–44 years	194.7	193.4196.1	199.2	197.9200.6	248.0	246.5249.5	287.9	286.3289.5	255.4	253.9256.9	237.3	235.9238.7	218.9	217.5220.2
45–54 years	395.9	394.0,397.9	392.4	390.5394.3	473.5	471.4475.6	531.0	528.7533.2	453.3	451.2455.4	411.9	409.9413.8	388.5	386.6390.5
55–64 years	886.7	883.9889.6	883.3	880.5886.1	1038.9	1035.91042.0	1117.1	1114.0,1120.3	992.1	989.1995.1	899.6	896.8902.5	853.8	851.0,856.6
65–74 years	1783.3	1778.61788.1	1764.6	1760.0,1769.3	2072.3	2067.32077.2	2151.3	2146.42156.3	1978.7	1974.0,1983.5	1809.6	1805.21814.1	1807.9	1803.41812.4
75–84 years	4386.1	4375.64396.5	4308.3	4298.14318.5	4997.0	4986.25007.8	5119.4	5108.45130.4	4708.2	4698.0,4718.4	4345.5	4336.0,4355.1	4472.9	4463.24482.6
85+ years	13,450.7	13,422.613478.8	13,228.6	13,200.913256.4	15,210.9	15,181.315240.5	15,743.3	15,711.515775.1	14,389.6	14,360.414418.8	14,285.8	14,256.114315.6	14,353.0	14,323.214382.8
Male	855.5	854.1857.0	846.7	845.3848.1	998.3	996.8999.8	1048.0	1046.41049.5	954.5	953.1956.0	884.3	882.9885.7	872.6	871.2874.0
Female	611.3	610.3612.4	602.8	601.7603.8	695.1	694.0,696.2	733.3	732.1734.4	666.1	665.0,667.2	632.9	631.8633.9	629.7	628.6630.7
Hispanic or Latino	524.1	521.8526.5	523.8	521.5526.1	723.6	720.9726.3	724.7	722.0,727.3	614.7	612.3617.0	559.2	556.9561.4	568.0	565.8570.3
Non-Hispanic American Indian or Alaska Native	790.8	778.8802.8	782.5	770.8794.3	1036.2	1022.91049.4	1109.2	1095.61122.9	947.9	935.6960.3	830.6	819.2842.0	807.3	796.1818.5
Non-Hispanic Asian	381.2	378.4384.1	372.8	370.02,375.59	457.7	454.7460.7	461.7	458.7464.8	417.5	414.8420.3	387.9	385.3390.5	396.9	394.3399.6
Non-Hispanic Black or African American	892.6	889.5895.7	884.0	881,887.03	1119.0	1115.71122.4	1118.0	1114.61121.4	1002.8	999.71006.0	924.4	921.4927.4	915.9	912.9918.9
Non-Hispanic Native Hawaiian or Other Pacific Islander	675.7	651.5699.9	679.0	655.6702.4	821.4	796.4846.3	924.3	898.5950.2	782.1	758.9805.2	730.1	708.2751.9	724.1	702.3745.9
Non-Hispanic White	748.7	747.7749.7	739.9	738.9740.9	834.8	833.7835.8	893.9	892.8895.1	822.2	821.1823.2	778.2	777.2779.2	765.9	764.9766.9
Non-Hispanic, More than one race	338.1	331.8344.4	326.5	320.5332.6	377.0	370.6383.3	406.0	399.6412.5	366.8	360.9372.6	352.2	346.6357.8	351.7	346.1357.3
Northeast	679.2	677.3681.1	669.5	667.6671.4	809.6	807.5811.7	768.3	766.3770.4	718.7	716.7720.6	677.0	675.1678.9	665.1	663.3667.0
Midwest	765.8	763.9767.7	757.7	755.8759.6	885.6	883.6887.6	904.8	902.8907.0	841.4	839.4843.4	789.1	787.2791.0	782.7	780.8784.6
South	767.6	766.2769.1	757.1	755.7758.5	877.0	875.4878.5	962.4	960.8964.0	852.7	851.3854.2	799.3	797.9800.7	792.1	790.7793.5
West	644.2	642.5645.9	640.4	638.7642.0	736.1	734.3737.9	806.3	804.4808.2	731.4	729.6733.1	689.4	687.7691.1	682.4	680.7684.1

Age-adjusted mortality rates for all groups except for age-groups, which are crude mortality rates (with 95 % CI). CI: Confidence Interval;

Trends in the rates of the top underlying causes of mortality during the study period are shown in Table 2 **and** Fig. 2. Diseases of the heart remained the top cause of mortality between 2019 and 2024, with age-adjusted mortality rates of 161.5 in 2019, peak age-adjusted mortality rates of 173.8 in 2021, and subsequent declines to age-adjusted mortality rates of 162.1 in 2024. Mortality rates from the second leading cause, malignancy, demonstrated more stable rates, with age-adjusted mortality rates of 146.2 in 2019, 144.1 in 2020, 146.6 in 2021, 142.3 in 2022,141.8 in 2023, and 143.2 in 2024. Mortality attributed to COVID-19 infection peaked in 2021 with age-adjusted mortality rates of 104.1 as the 3rd leading cause of death and then dropped to 44.5 as the 4th leading cause of death in 2022, 11.9 as the 11th leading cause in 2023, and 7.5 as the 15th leading cause of death in 2024 **(**Fig. 2**,** Table 2**)**.

**Table 2:** Top 15 underlying causes of death per year in the United States, expressed as Crude Mortality Rates/Age-Adjusted Mortality Rates per 100,000 population, obtained from CDC WONDER from 2018 to 2024.

	2018		2019		2020		2021		2022		2023		Provisional 2024
Diseases	Age-Adjusted Mortality Rate (95% CI)	Diseases	Age-Adjusted Mortality Rate (95% CI)	Diseases	Age-Adjusted Mortality Rate (95% CI)	Diseases	Age-Adjusted Mortality Rate (95% CI)	Diseases	Age-Adjusted Mortality Rate (95% CI)	Diseases	Age-Adjusted Mortality Rate (95% CI)	Diseases	Age-Adjusted Mortality Rate (95% CI)
Diseases of heart	163.6 (163.2,164.0)	Diseases of heart	161.5 (161.1,161.9)	Diseases of heart	168.2 (167.8,168.6)	Diseases of heart	173.8 (173.4,174.2)	Diseases of heart	167.2 (166.8,167.6)	Diseases of heart	162.1 (161.7,162.5)	Diseases of heart	162.1 (161.7,162.5)
Malignant neoplasm	149.1 (148.7,1 49.5)	Malignant neoplasms	146.2 (145.8,146.5)	Malignant neoplasms	144.1 (143.8,144.5)	Malignant neoplasms	146.6 (146.2,146.9)	Malignant neoplasms	142.3 (141.9,142.7)	Malignant neoplasms	141.8 (141.4,142.1)	Malignant neoplasms	143.2 (142.8,143.5)
Accidents (unintentional injuries)	48.0 (47.8,48.2)	Accidents (unintentional injuries)	49.3 (49.1,49.5)	COVID,19	85.0 (84.7,85.3)	COVID,19	104.1 (103.8,104.4)	Accidents (unintentional injuries)	64.0 (63.8,64.3)	Accidents (unintentional injuries)	62.3 (62.1,62.6	Accidents (unintentional injuries)	43.0 (42.8,43.2)
Chronic lower respiratory diseases	39.7 (39.5,39.9)	Chronic lower respiratory diseases	38.2 (38.0,38.4)	Accidents (unintentional injuries)	57.6 (57.3,57.9)	Accidents (unintentional injuries)	64.7 (64.4,65.0)	COVID,19	44.5 (44.3,44.7)	Cerebrovascular diseases	39.0 (38.8,39.2)	Cerebrovascular diseases	39.9 (39.7,40.1)
Cerebrovascular diseases	37.1 (36.9,37.3)	Cerebrovascular diseases	37.0 (36.8,37.2)	Cerebrovascular diseases	38.8 (38.7,39.0)	Cerebrovascular diseases	41.1 (40.9,41.3)	Cerebrovascular diseases	39.5 (39.3,39.7)	Chronic lower respiratory diseases	33.4 (33.3,33.6)	Chronic lower respiratory diseases	33.4 (33.3,33.6)
Alzheimer disease	30.5 (30.4,30.7)	Alzheimer disease	29.9 (29.7,30.0)	Chronic lower respiratory diseases	36.4 (36.2,36.6)	Chronic lower respiratory diseases	34.7 (34.5,34.9)	Chronic lower respiratory diseases	34.3 (34.1,34.5)	Alzheimer disease	27.8 (27.6,27.9)	Alzheimer disease	28.2 (28.0,28.3)
Diabetes mellitus	21.4 (21.2,21.5)	Diabetes mellitus	21.6 (21.4,21.7)	Alzheimer disease	32.5 (32.3,32.6)	Alzheimer disease	31.0 (30.8,31.1)	Alzheimer disease	28.9 (28.7,29.1)	Diabetes mellitus	22.4 (22.3,22.5)	Diabetes mellitus	22.2 (22.0,22.3)
Influenza and pneumonia	14.9 (14.8,15.0)	Intentional self,harm (suicide)	13.9 (13.8,14.1)	Diabetes mellitus	24.8 (24.7,25.0)	Diabetes mellitus	25.4 (25.3,25.6)	Diabetes mellitus	24.1 (23.9,24.2)	Intentional self,harm (suicide)	14.1 (14.0,14.3)	Nephritis, nephrotic syndrome and nephrosis	13.0 (12.9,13.1)
Intentional self,harm (suicide)	14.2 (14.1,14.4)	Nephritis, nephrotic syndrome and nephrosis	12.7 (12.6,12.8)	Intentional self,harm (suicide)	13.5 (13.3,13.7)	Chronic liver disease and cirrhosis	14.5 (14.3,14.6)	Intentional self,harm (suicide)	14.2 (14.1,14.4)	Nephritis, nephrotic syndrome and nephrosis	13.1 (13.0,13.2)	Chronic liver disease and cirrhosis	12.9 (12.8,13.1)
Nephritis, nephrotic syndrome and nephrosis	12.9 (12.8,13.0)	Influenza and pneumonia	12.3 (12.2,12.4)	Chronic liver disease and cirrhosis	13.3 (13.1,13.4)	Intentional self,harm (suicide)	14.1 (14.0,14.2)	Nephritis, nephrotic syndrome and nephrosis	13.8 (13.7,13.9)	Chronic liver disease and cirrhosis	13.0 (12.9,13.1)	Influenza and pneumonia	11.5 (11.4,11.6)
Chronic liver disease and cirrhosis	11.1 (11.0,11.2)	Chronic liver disease and cirrhosis	11.3 (11.2,11.5)	Influenza and pneumonia	13.1 (12.9,13.2)	Nephritis, nephrotic syndrome and nephrosis	13.6 (13.4,13.7)	Chronic liver disease and cirrhosis	13.8 (13.7,14.0)	COVID,19	11.9 (11.7,12.0)	Intentional self,harm (suicide)	11.1 (11.0,11.2)
Septicemia	10.3 (10.2,10.4)	Septicemia	9.5 (9.4,9.6)	Nephritis, nephrotic syndrome and nephrosis	12.7 (12.6,12.8)	Essential hypertension and hypertensive renal disease	10.7 (10.6,10.8)	Influenza and pneumonia	11.3 (11.2,11.4)	Influenza and pneumonia	10.9 (10.8,11.0)	Essential hypertension and hypertensive renal disease	10.1 (10.0,10.1)
Essential hypertension and hypertensive renal disease	8.9 (8.8,9.0)	Essential hypertension and hypertensive renal disease	8.9 (8.8,9.0)	Essential hypertension and hypertensive renal disease	10.1 (10.0,10.2)	Influenza and pneumonia	10.5 (10.4,10.6)	Essential hypertension and hypertensive renal disease	10.3 (10.2,10.4)	Essential hypertension and hypertensive renal disease	10.1 (10.0,10.2)	Parkinson disease	9.7 (9.6,9.8)
Parkinson disease	8.7 (8.6,8.8)	Parkinson disease	8.8 (8.7,8.9)	Parkinson disease	9.9 (9.8,10.0)	Septicemia	10.2 (10.1,10.3)	Septicemia	10.1 (10.0,10.2)	Septicemia	9.9 (9.8,10.0)	Septicemia	9.5 (9.4,9.6)
Pneumonitis due to solids and liquids	4.8 (4.7,4.9)	Pneumonitis due to solids and liquids	4.7 (4.6,4. 8)	Septicemia	9.7 (9.6,9.8)	Parkinson disease	9.8 (9.7,9.9)	Parkinson disease	9.5 (9.4,9.6)	Parkinson disease	9.5 (9.4,9.6)	COVID,19	7.5 (7.4,7.6)

Age-Adjusted Mortality rates with 95 % Confidence Interval (CI).

#Diseases of heart (I00,I09,I11,I13,I20,I51), #Malignant neoplasms (C00,C97), #Accidents (unintentional injuries) (V01,X59,Y85,Y86), #Chronic lower respiratory diseases (J40,J47), #Cerebrovascular diseases (I60,I69), #Alzheimer's disease (G30), #Diabetes mellitus (E10,E14), #Influenza and pneumonia (J09,J18), #Nephritis, nephrotic syndrome and nephrosis (N00,N07,N17,N19,N25,N27), #Intentional self,harm (suicide) (*U03,X60,X84,Y87.0), #Chronic liver disease and cirrhosis, #Septicemia (A40,A41) (K70,K73,K74), #Essential hypertension and hypertensive renal disease (I10,I12,I15), #Parkinson's disease (G20,G21), #Pneumonitis due to solids and liquids (J69), #COVID,19 (U07.1).

**Fig. 2:**
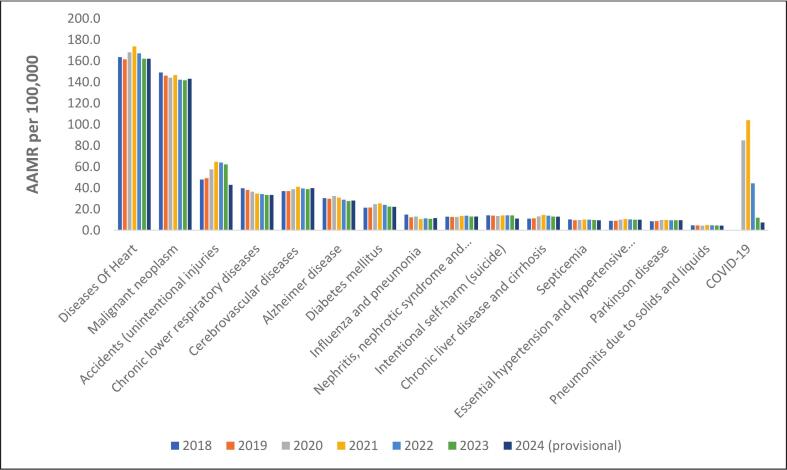
Underlying cause of death - Fifteen leading causes of death for each year, 2018–2024, in the United States obtained from the Center for Disease Control and Prevention Wide-Ranging Online Data for Epidemiologic Research.

## 4 Discussion

Our description of United States mortality trends surrounding the peak of the COVID pandemic, including trends based on the latest available 2024 provisional mortality data, demonstrates several important findings. Since peaking in 2021, all-cause mortality has continued to decline in subsequent years, including a decline between 2023 and provisional 2024 data. However, 2024 provisional all-cause mortality remains above pre-pandemic rates. Similar declines in all-cause mortality from the 2021 trends were noted among both sexes, among racial and ethnic groups, and among some age groups. Evaluation of trends for top individual causes of mortality demonstrate different trends among the leading causes of mortality, with some causing remaining above pre-pandemic rates while other causes returning to or falling below pre-pandemic rates. Mortality attributed to COVID declines from the 3rd leading cause of death in 2021 to the 15th leading cause in provisional 2024 data. These data highlight improving mortality trends since the peak of the COVID pandemic but demonstrate ongoing elevations in all-cause and many individual causes of mortality compared to pre-pandemic rates.

The provisional 2024 data presented here expand on prior analyses of the mortality rates and trends in the earlier phases of the COVID pandemic (Ahmad et al., 2024; Bor et al., 2025; Minhas et al., 2024; Murphy et al., 2024; Ahmad et al., 2023; Walkowiak et al., 2023; Kobo et al., 2023). Previous publications on trends through 2023 noted declines in all-cause mortality from the 2021 peak (Ahmad et al., 2024; Minhas et al., 2024; Murphy et al., 2024). By updating all-cause mortality with provisional 2024 data, we demonstrate that while all-cause mortality in 2024 continued to decline compared to 2023, mortality in 2024 remains above pre-pandemic values. Ongoing elevated all-cause mortality in provisional 2024 mortality data may be due to various factors. Although mortality from COVID has decreased, COVID remains the 15th leading cause of death and the sequalae of COVID infection may lead to greater morbidity and mortality from other conditions. Ongoing all-cause mortality rates above pre-pandemic baseline may nevertheless be attenuated by trends in causes of death from top individual causes of death, such as diseases of the heart or malignant neoplasms, where mortality appears to have returned to baseline or potentially dipped below pre-pandemic rates. Variable effects of the pandemic on individual causes of mortality may be multifactorial, including due to resumption of standard medical care with the waning of the direct effects of the pandemic. Additionally, some individuals who would have died from other causes of death in subsequent years may have prematurely died in the early phases of the COVID pandemic, a phenomenon terms “harvesting effect,” (Walkowiak et al., 2023) which may explain why rates for conditions such as malignant neoplasms, chronic lower respiratory disease, or Alzheimer's disease now appear to be below pre-pandemic baseline. Analyses in subsequent years will therefore be needed to determine when mortality rates return to baseline, as current projections vary and have important implications for public health prognostication in the US (*The Demographic Outlook*: 2025 to 2055. Congressional Budget Office. https://www.cbo.gov/publication/61164#:∼:text=In%20CBO's%20projections%2C%20the%20population, 2025; *The future of excess mortality after COVID-19. Swiss Re Institute. https://www.swissre.com/institute/research/topics-and-risk-dialogues/health-and-longevity/covid-19-pandemic-synonymous-excess-mortality.html*, 2025). The United States data presented in our analysis parallels mortality trends in other world regions, including Europe, where most countries in Western and Northern Europe continued to report excess mortality in 2022 and 2023 compared to pre-pandemic baseline (Pizzato et al., 2024; Devanathan et al., 2025; Rancourt et al., 2024). Future comparative studies between the United States and other countries or regions may be beneficial to assess the potential effects of different public health interventions on outcomes.

Our results also highlight ongoing trends in provisional 2024 mortality data among important cohorts based on sex, age, and race and ethnicity. We demonstrate that provisional 2024 all-cause mortality remained above pre-pandemic baseline in both men and women. Similar findings were noted among race and ethnicity groups. Absolute all-cause mortality rates in 2024 remained highest in Non-Hisbapic Black or African American individuals. While mortality trends based on race and ethnicity cohorts during the COVID pandemic have been previously reported, we highlight the ongoing public health consequences of the pandemic across racial and ethnic groups noted in provisional 2024 mortality data. Further surveillance of mortality among racial and ethnicity cohorts will be needed to identify potential disparities which may result in slower mortality improvement in certain populations.

Our results also highlight prominent differences in all-cause mortality trends among age groups between pre-pandemic 2019 data and provisional 2024 mortality data. Mortality for adults ≥65 years of age remained higher in provisional 2024 data compared to 2019 data, with findings most prominent for patients ≥85 years of age. Residual mortality elevation in older adults may be attributable to the residual effects of COVID infection on morbidity and mortality, which may be greatest in older individuals. Specifically, older individuals have a greater burden of chronic conditions and lower immune system responsiveness (Abidin et al., 2025), which compound risks of a COVID compared to younger cohorts. These findings suggest that COVID related mortality in older adults may remain elevated as long the virus continues to circulate. On the other hand, analyses in several younger cohorts of middle aged and young adults demonstrate that provisional 2024 mortality returned to or potentially fell below the 2019 pre-pandemic rates. There may be several explanations for these findings in middle aged and younger adults. Causes of death that tend to affect younger cohorts, including suicide and accidents, had prominent declines in 2024 data compared to both the pandemic peak and pre-pandemic baseline. Working aged individuals may have also been positively affected by socioeconomic factors such as greater access to health insurance due to Medicaid expansion which surrounded the peak of the COVID era (*Healthcare Insurance Coverage, Affordability of Coverage, and Access to Care, 2021–2024. https://aspe.hhs.gov/sites/default/files/documents/9a943f1b8f8d3872fc3d82b02d0df466/coverage-access-2021-2024.pdf*, 2025; *Key Facts about the Uninsured Population. https://www.kff.org/uninsured/issue-brief/key-facts-about-the-uninsured-population*/, 2025), although these potential factors or other differences require further investigation. Future surveillance of mortality rates among younger cohorts who are not yet eligible for Medicare, particularly in an era of potential changes to Medicaid enrollment criteria, will be required.

There are limitations to our analysis. The data from 2024 are provisional, utilize population denominator of previous year, and will be updated with future iterations of data releases within Center for Disease Control and Prevention Wide-Ranging Online Data for Epidemiologic Research. The provisional nature of the 2024 data used in this analysis implies the potential for changes to the 2024 data once the data are finalized in the subsequent year. Determination of causes of death rely on the accuracy of causes filled out on the death certificate. The leading cause of death data represents only the underlying cause of death, which may underestimate the burden of particular causes of death, especially when multiple causes may play key roles in fatal outcomes (Minhas et al., 2025). Other important patient characteristics, such as socioeconomic factors are not available in this dataset. Analyses of cohorts, including those based on age, race and ethnicity, and causes of death are limited to the classifications available within Center for Disease Control and Prevention Wide-Ranging Online Data for Epidemiologic Research. Our analyses do not account for unmeasured confounding factors such as changes in population health behaviors or particular public health interventions.

## 5 Conclusions

In conclusion, provisional 2024 all-cause mortality estimates remain above pre-pandemic mortality rates in the United States. Mortality from COVID decreased from the 3rd leading cause of death in 2021 to the 15th leading cause in 2024. All-cause mortality rates in 2024 remained above pre-pandemic baseline for both sexes, all race and ethnicity groups, and for some age groups. There were differences in mortality trends noted among the top individual causes of death, with mortality from some conditions in 2024 remaining above pre-pandemic rates and mortality from other conditions returning to, or dropping below, the pre-pandemic baseline. These results highlight the ongoing mortality effects of the COVID pandemic and demonstrate important differences in mortality trends among different age groups and among top individual causes of death.

## CRediT authorship contribution statement

**Shehroze Tabassum:** Writing – review & editing, Writing – original draft, Formal analysis, Conceptualization. **Faraz Azhar:** Writing – review & editing. **Abdul Mannan Khan Minhas:** Writing – review & editing, Writing – original draft, Visualization, Formal analysis. **Dmitry Abramov:** Writing – review & editing, Writing – original draft, Supervision.

## Ethical considerations

No ethical approval was required for this study design, as all data were obtained from publicly available sources.

## Funding

This research received no funding from any agency in the public, commercial, or not-for-profit sectors.

## Declaration of competing interest

The authors declare that they have no known competing financial interests or personal relationships that could have appeared to influence the work reported in this paper.

## Data Availability

data are publically available

## References

[bb0005] Abidin Z. ul, Thirumalareddy J., Gupta J.S., Abdul Jabbar A.B. (2025). Disparities in COVID-19 mortality in the United States, 2020–2023. BMC Public Health.

[bb0010] Ahmad F.B., Anderson R.N., Knight K., Rossen L.M., Sutton P.D. (2021). Advancements in the National Vital Statistics System to meet the real-time data needs of a pandemic. Am. J. Public Health.

[bb0015] Ahmad F.B., Cisewski J.A., Anderson R.N. (2024). Leading causes of death in the US, 2019-2023. JAMA.

[bb0020] Ahmad F.B., Cisewski J.A., Xu J., Anderson R.N. (2023). Provisional mortality data—United States, 2022. Morb. Mortal. Wkly. Rep..

[bb0025] Anderson R.N., Rosenberg H.M. (1998).

[bb0030] Bor J., Raquib R.V., Wrigley-Field E., Woolhandler S., Himmelstein D.U., Stokes A.C. (2025). Excess US Deaths Before, during, and after the COVID-19 Pandemic. American Medical Association. JAMA Health Forum.

[bb0035] Devanathan G., Chua P.L., Nomura S., Ng C.F.S., Hossain N., Eguchi A., Hashizume M. (2025). Excess mortality during and after the COVID-19 emergency in Japan: a two-stage interrupted time-series design. *BMJ*. Public Health.

[bb0040] (2025). Healthcare Insurance Coverage, Affordability of Coverage, and Access to Care, 2021–2024. https://aspe.hhs.gov/sites/default/files/documents/9a943f1b8f8d3872fc3d82b02d0df466/coverage-access-2021-2024.pdf.

[bb0045] (2025). Key Facts about the Uninsured Population. https://www.kff.org/uninsured/issue-brief/key-facts-about-the-uninsured-population/.

[bb0050] Kobo O., Abramov D., Fudim M., Sharma G., Bang V., Deshpande A., Wadhera R.K., Mamas M.A. (2023). Has the first year of the COVID-19 pandemic reversed the trends in CV mortality between 1999 and 2019 in the United States?. Eur. Heart J.-Qual. Care Clin. Outcomes..

[bb0055] Minhas A.M.K., Fudim M., Michos E.D., Abramov D. (2024). Has mortality in the United States returned to pre-pandemic levels? An analysis of provisional 2023 data. J. Intern. Med..

[bb0060] Minhas A.M.K., Sperling L.S., Al-Kindi S., Abramov D. (2025). Underlying and contributing causes of mortality from CDC WONDER—insights for researchers. Am. Heart J. Plus Cardiol. Res. Pract..

[bb0065] Murphy S.L., Kochanek K.D., Xu J., Arias E. (2024).

[bb0070] Pizzato M., Gerli A.G., La Vecchia C., Alicandro G. (2024). Impact of COVID-19 on total excess mortality and geographic disparities in Europe, 2020–2023: a spatio-temporal analysis. Lancet Reg. Heal..

[bb0075] (2024). Provisional Mortality Statistics by Multiple Cause of Death and by Single Race, for 2018 through Present. https://wonder.cdc.gov/wonder/help/mcd-provisional.html.

[bb0080] Rancourt D.G., Hickey J., Linard C. (2024).

[bb0085] (2025). The Demographic Outlook: 2025 to 2055. Congressional Budget Office. https://www.cbo.gov/publication/61164#:∼:text=In%20CBO's%20projections%2C%20the%20population.

[bb0090] (2025). The future of excess mortality after COVID-19. Swiss Re Institute. https://www.swissre.com/institute/research/topics-and-risk-dialogues/health-and-longevity/covid-19-pandemic-synonymous-excess-mortality.html.

[bb0095] Vital Statistics of United States (1999).

[bb0100] Walkowiak M., Domaradzki J., Walkowiak D. (2023). Unmasking the COVID-19 pandemic prevention gains: excess mortality reversal in 2022. Public Health.

